# Cardiac Ablation of *Rheb1* Induces Impaired Heart Growth, Endoplasmic Reticulum-Associated Apoptosis and Heart Failure in Infant Mice

**DOI:** 10.3390/ijms141224380

**Published:** 2013-12-13

**Authors:** Yunshan Cao, Lichan Tao, Shutong Shen, Junjie Xiao, Hang Wu, Beibei Li, Xiangqi Wu, Wen Luo, Qi Xiao, Xiaoshan Hu, Hailang Liu, Junwei Nie, Shuangshuang Lu, Baiyin Yuan, Zhonglin Han, Bo Xiao, Zhongzhou Yang, Xinli Li

**Affiliations:** 1 Department of Cardiology, The First Affiliated Hospital with Nanjing Medical University, Nanjing 210029, China; E-Mails: yunshancao@126.com (Y.C.); sherry0019@126.com (L.T.); shenst1989@gmail.com (S.S.); junjiexiao@shu.edu.cn (J.X.); wh58806@163.com (H.W.); hello_beibei@163.com (B.L.); wuxiangqi2007@sina.com (X.W.); lhl2ny@yeah.net (H.L.); njzyl165@126.com (Z.H.); 2 MOE Key Laboratory of Model Animal for Disease Study, Model Animal Research Center, Nanjing University, Nanjing 210061, China; E-Mails: luowen2345@126.com (W.L.); mac860622@gmail.com (Q.X.); njhuxs@126.com (X.H.); icbm2000@126.com (J.N.); luss@nicemice.cn (S.L.); baiyinyuan@gmail.com (B.Y.); 3 Regeneration Lab and Experimental Center of Life sciences, School of Life Sciences, Shanghai University, Shanghai 200444, China; 4 The State Key Laboratory of Biotherapy, West China Hospital, Sichuan University, Chengdu 610041, China; E-Mail: bxiao365@yahoo.com

**Keywords:** Rheb1, heart growth, infant heart failure, mTORC1, ER

## Abstract

Ras homologue enriched in brain 1 (Rheb1) plays an important role in a variety of cellular processes. In this study, we investigate the role of Rheb1 in the post-natal heart. We found that deletion of the gene responsible for production of Rheb1 from cardiomyocytes of post-natal mice resulted in malignant arrhythmias, heart failure, and premature death of these mice. In addition, heart growth impairment, aberrant metabolism relative gene expression, and increased cardiomyocyte apoptosis were observed in *Rheb1*-knockout mice prior to the development of heart failure and arrhythmias. Also, protein kinase B (PKB/Akt) signaling was enhanced in *Rheb1*-knockout mice, and removal of phosphatase and tensin homolog (*Pten*) significantly prolonged the survival of *Rheb1-*knockouts. Furthermore, signaling via the mammalian target of rapamycin complex 1 (mTORC1) was abolished and C/EBP homologous protein (CHOP) and phosphorylation levels of c-Jun *N*-terminal kinase (JNK) were increased in *Rheb1* mutant mice. In conclusion, this study demonstrates that Rheb1 is important for maintaining cardiac function in post-natal mice via regulation of mTORC1 activity and stress on the endoplasmic reticulum. Moreover, activation of Akt signaling helps to improve the survival of mice with advanced heart failure. Thus, this study provides direct evidence that Rheb1 performs multiple important functions in the heart of the post-natal mouse. Enhancing Akt activity improves the survival of infant mice with advanced heart failure.

## 1. Introduction

Ras homologue enriched in brain 1 (Rheb1) is a small GTPase that regulates cell growth, cell fate, energy metabolism, and mTORC1 activation *in vitro* [1–3]. Recent studies have demonstrated that Rheb1 plays a pivotal role in post-natal brain development [4] and embryonic cardiovascular development [5]. It has been reported that Rheb1 activates protein synthesis and growth in rat ventricular cardiomyocytes *in vitro* [3]. Our recent study, which investigated the effects of half-knock out of *Rheb1* in adult mice in myocardial infarction (MI) and hypertrophic cardiomyopathy (HCM), showed that reduction of Rheb1-mTORC1 signaling protects against pathological heart remodeling in MI and HCM [6]. However, little is known about the role that Rheb1 plays in mediating the function of the post-natal heart *in vivo*.

Here we report that complete knockout of the *Rheb1* gene (*Rheb1* cKO) results in malignant arrhythmias, heart failure and premature death in post-natal mice. Heart growth impairment and increased cardiomyocyte apoptosis have already been observed in *Rheb1* cKO mice prior to the development of heart failure and arrhythmias. Moreover, mTORC1 activity was abolished and C/EBP homologous protein (CHOP) and phosphorylation levels of c-Jun *N*-terminal kinase (JNK) were increased in *Rheb1* mutant mice. Also, Akt signaling was enhanced in *Rheb1*-deletion mice. Removal of phosphatase and tensin homolog (*Pten*) significantly prolonged the survival of *Rheb1*-deletion mice, indicating that the activation of Akt in *Rheb1*-deletion mice represents a protective adaptive compensatory response.

During the preparation of this manuscript, the phenotype of *Rheb1*-deletion mice was reported by the Otsu group; our results support the findings of Otsu *et al.* [7], and build on their work in a number of ways. First, we are able to present a more comprehensive *Rheb1* cKO mouse phenotype; specifically, we show that *Rheb1* cKO mice have altered metabolic genes, arrhythmias and increased cardiac cell apoptosis. Second, our work yields new insights into the crosstalk between Rheb1-mTOR signaling and ER stress signaling. Third, we provide evidence that Akt hyperphosphorylation in *Rheb1*-deletion mice may be protective, a finding that may have significant clinical implications, particularly for patients with heart failure.

## 2. Results and Discussion

### 2.1. Results

#### 2.1.1. Development of *Rheb1* cKO Mouse

**Figure 1.**
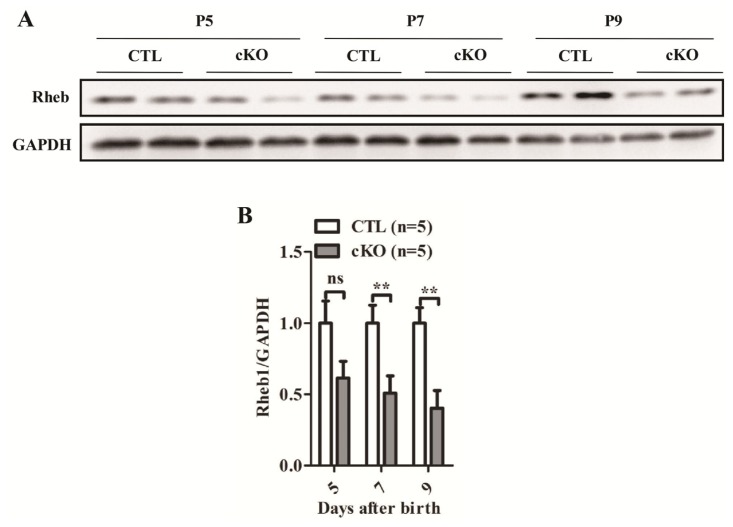
(**A**) Detection of deletion efficiency of *Rheb1* by Western blot at postnatal day 5, 7, and 9, respectively; and (**B**) Quantification of (**A**) (*n* = 5). Abbreviations: CTL, Control group (*Rheb1^F^*^/^*^F^*); cKO, cardiac knockout group (*Rheb1^F^*^/^*^F^*; *αMHC-Cre*); **p < 0.01, cKO *versus* CTL.

To investigate the role of Rheb1 in the mouse heart, we generated cardiac-specific *Rheb1* cKO mice. *Rheb1^F^*^/^*^F^* mice were crossed with *αMHC-Cre* mice to obtain *Rheb1^F^*^/^*^F^*; *αMHC-Cre* (cKO) mice. *Rheb1^F^*^/^*^F^* littermates were used as controls (CTL). *Rheb1* cKO mice were born at a *Mendelian* ratio, and were indistinguishable from control littermates (Supplementary Table S1). Western blot analysis confirmed that Rheb1 protein levels were significantly reduced in *Rheb1* cKO mice (Figure 1A,B).

#### 2.1.2. Loss of *Rheb1* Causes Heart Failure, Malignant Arrhythmias and Premature Death at Infant Stage

**Figure 2.**
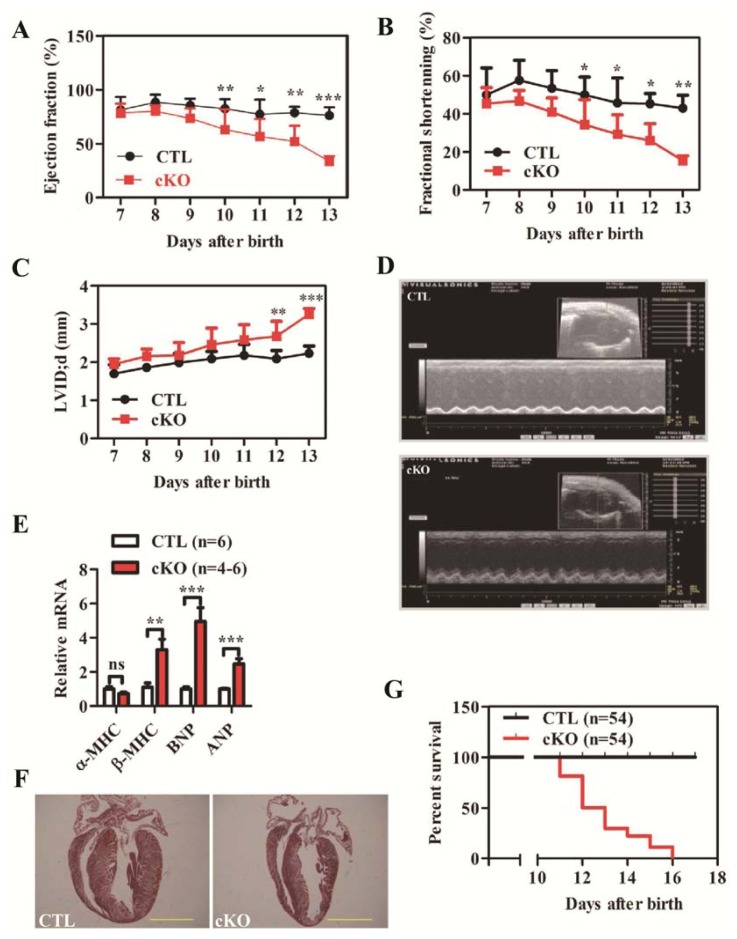
(**A**–**C**) Comparison of Ejection fraction (EF), Fractional shortening (FS) and LVIDd between cKO and CTL at different stages (*n* = 8 at days 7–11, *n* = 5 at day 12 and *n* = 3 at day 13); (**D**) Representative echocardiography images at postnatal day 12; (**E**) qPCR detection of heart remodeling markers including *αMHC*, *βMHC*, *BNP* and *ANP* (*n* = 4–6); (**F**) Masson’s staining (Scale bar = 1.5 mm); and (**G**) Survival analysis (*n* = 54). Abbreviations: CTL, Control group (*Rheb1^F^*^/^*^F^*); cKO, cardiac knockout group (*Rheb1^F^*^/^*^F^*; *αMHC-Cre*); LVID; d, LV internal diameter in diastole; *αMHC*, α-myosin heavy chain; *βMHC*, β-myosin heavy chain; *BNP*, brain natriuretic peptide; *ANP*, atrial natriuretic peptide. *p < 0.05; **p < 0.01; and ***p < 0.001, cKO *versus* CTL.

We analyzed heart contractility using echocardiography (Echo) in control (CTL) and *Rheb1* cKO mice. *Rheb1* cKO mice exhibit reduced contractility and cardiac dilatation at post-natal days 10 and 12, respectively (Figure 2A–D; Supplementary Table S2). Expression levels of β-myosin heavy chain (β-*MHC*), brain natriuretic peptide (*BNP*), and atrial natriuretic peptide (*ANP*) were all significantly increased in *Rheb1* cKO mice compared to controls, suggesting that pathological heart remodeling occurred in these mice (Figure 2E). However, Masson’s staining failed to detect any apparent fibrosis in *Rheb1* cKO mice (Figure 2F).

**Figure 3.**
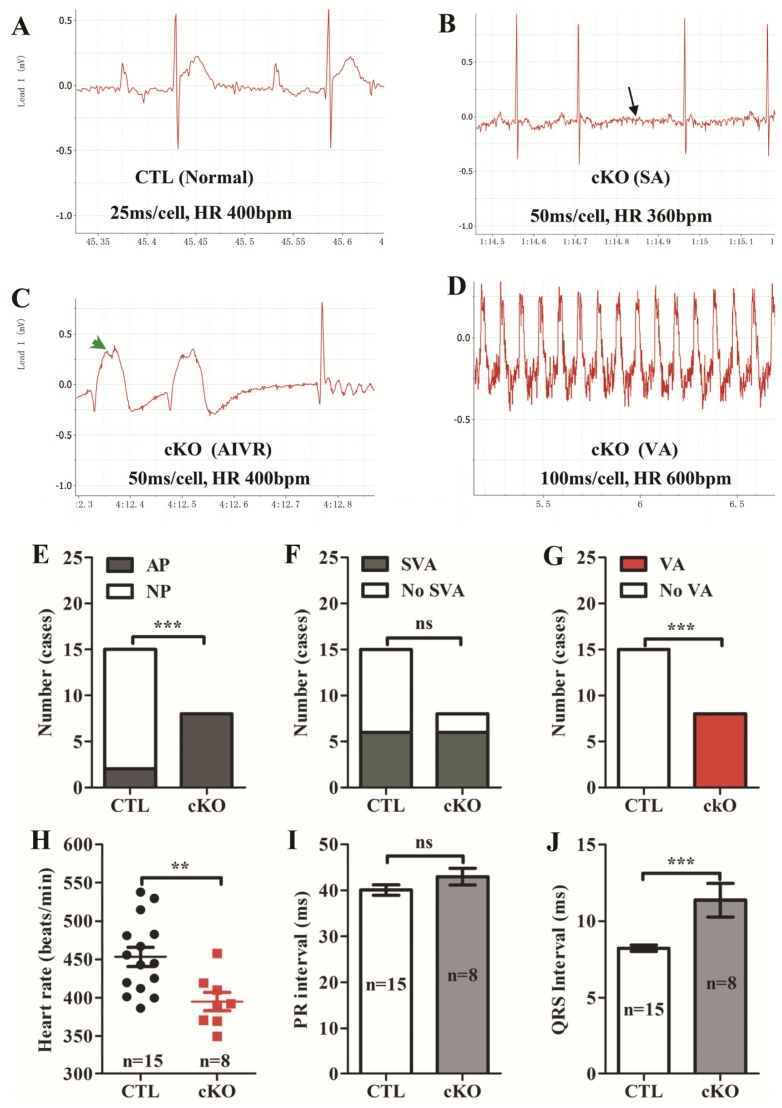
(**A**) ECG recording from a CTL mouse; (**B**) Transient sinus arrest was recorded in cKO (See black arrow); (**C**) Accelerated idioventricular rhythm (AIVR) (See green arrowhead) in a cKO case; (**D**) Ventricular tachycardia (VA) was recorded in cKO; and (**E**–**J**) Quantification of arrhythmias and heart rates (*n* = 8–15). Abbreviations: CTL, Control group (*Rheb1^F^*^/^*^F^*); cKO, cardiac knockout group (*Rheb1^F^*^/^*^F^*; *αMHC-Cre*); SA, Sinus arrest; AP, Abnormal P wave including lower P wave, flattened P wave, and P wave disappearance; NP, Normal P wave; SVA, Supra-ventricular arrhythmias including sinus arrest, atrioventricular block; VA, Ventricular arrhythmias including ventricular tachycardia and accelerated idioventricular rhythm; HR, heart rate. **p < 0.01; and ***p < 0.001, cKO *versus* CTL.

Electrocardiography recordings showed that electrical abnormalities existed in *Rheb1* mutant mice (Figure 3A–I), for example, flattened P waves occurred more frequently in *Rheb1* cKO mice than in CTL mice (Figure 3E). In addition, heart rate was slower in *Rheb1* mutant mice than in CTL mice (Figure 3H) and the duration of the QRS interval was prolonged compared to CTLs (Figure 3J). Ventricular arrhythmias only occurred in *Rheb1* mutant mice (Figure 3G), however, no significant differences in rates of supra-ventricular arrhythmias (SVA) or in PR interval duration were observed between *Rheb1* mutants and CTLs (Figure 3F,I). Survival analysis showed that *Rheb1* cKO mice started to die at post-natal day 11, and that all mice died before post-natal day 16 (Figure 2G).

Next, we tested whether ablation of *Rheb1* at post-natal day 9 affected heart growth, energy metabolism and cardiomyocyte survival when mouse cardiac function was normal.

#### 2.1.3. Cardiac Deletion of *Rheb1* Results in Abnormal Heart Growth and Retarded Cardiomyocyte Size

**Figure 4.**
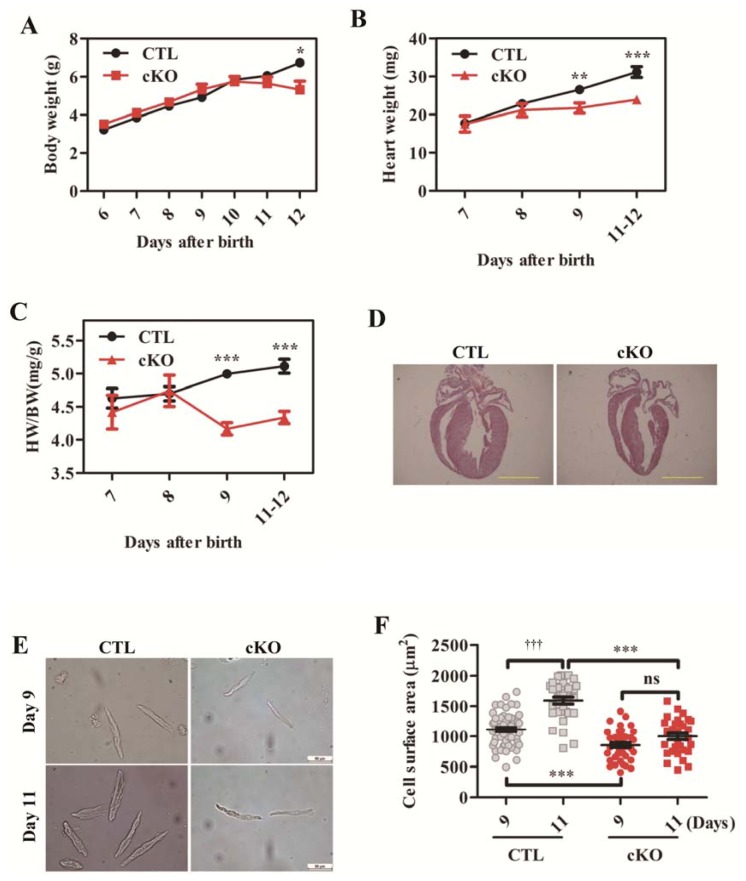
(**A**) Body weight (*n* = 3–53); (**B**) Heart weight (*n* = 4–24); (**C**) Ratio of heart weight to body weight (*n* = 4–24); (**D**) H&E staining (Scale bar = 1.5 mm); (**E**) Isolated cardiomyocytes at post-natal days 9 and 11 (Scale bar = 50 μm); and (**F**) Quantification of cardiomyocyte surface area in (**E**) (*n* = 5). Abbreviations: CTL, Control group (*Rheb1^F^*^/^*^F^*); cKO, cardiac knockout group (*Rheb1^F^*^/^*^F^*; *αMHC-Cre*); HW, heart weight; BW, body weight; H&E, Hematoxylin and eosin. *p < 0.05; **p < 0.01; and ***p < 0.001, cKO *versus* CTL; ^†††^*p* < 0.001, day 11 *versus* day 9 in CTL.

In control mice, body weight and heart weight increased consistently with age (Figure 4A,B). However, in *Rheb1* cKO mice, heart growth was significantly retarded and heart-to-body weight ratio was decreased prior to the onset of significant cardiac dysfunction at post-natal day 9 (Figure 4B–D). The body weight of *Rheb1* cKO mice started to decrease at postnatal day 11 (Figure 4A). Histological analysis showed that the ventricles of *rheb* 1 mutant mice had thinner walls and larger volume than the ventricles of control mice (Figure 4D). The cardiomyocyte size was smaller in *Rheb1* cKO mice than in controls as well (Figure 4E–F). Taken together, these results indicate that Rheb1 is essential for post-natal cardiomyocyte growth, and plays a pivotal role in maintaining normal cardiac function after birth.

#### 2.1.4. *Rheb1* Deletion Results in a Disrupted Expression Profile of Metabolic Genes and Increased Cardiomyocyte Apoptosis, but Does not Affect Autophagy

**Figure 5.**
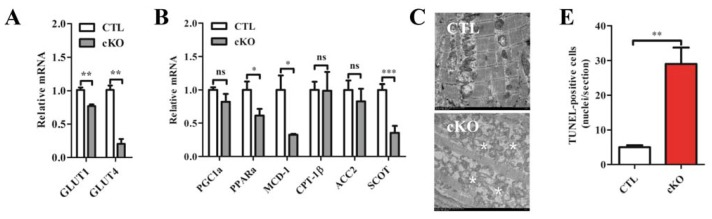

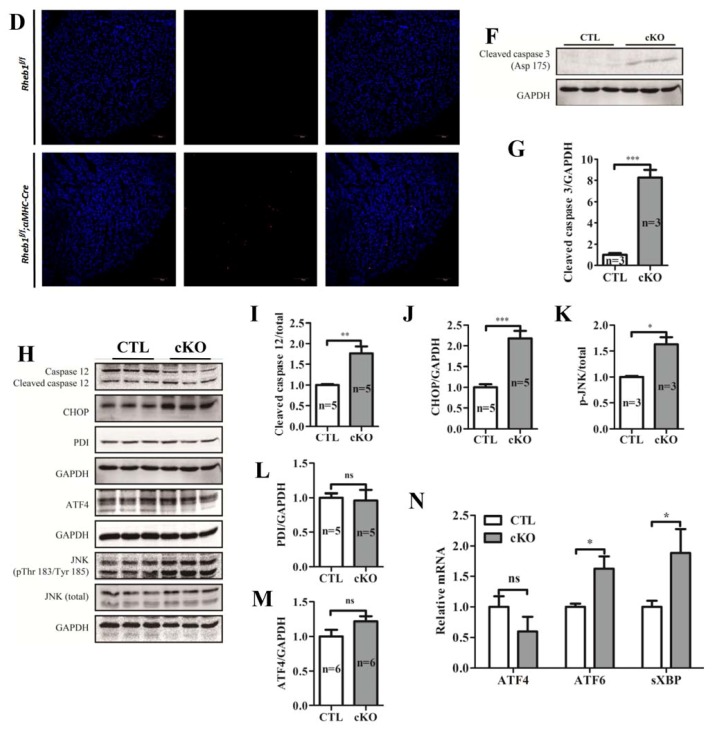
(**A**) Levels of expression of genes involved in glucose metabolism by qPCR at post-natal day 9 (*n* = 4–6); (**B**) Levels of expression of genes involved in fatty acid metabolism by qPCR at post-natal day 9 (*n* = 4–6); (**C**) Transmission electron microscope (TEM) analysis. Dysfunctional mitochondria were detected (indicated by asterisk) in cKO; (**D**) TUNEL staining. Red indicates TUNEL positive; blue indicates DAPI staining of nuclei. Original magnification: ×40 (Scale bar = 60 μm); (**E**) Quantification of (**D**) (*n* = 3). (**F**) Western blot analysis of cleaved caspase 3; (**G**) Quantification of (F); (**H**) Western blot analysis of ER-associated signaling pathways in CTL and *Rheb1* cKO mice at post-natal day 9; (**I**–**M**) Quantification of (H), (*n* = 3–6); and (**N**) Detection of ER sensors at post-natal day 9 by qPCR (*n* = 4–6). Abbreviations: CTL, Control group (*Rheb1^F^*^/^*^F^*); cKO, cardiac knockout group (*Rheb1^F^*^/^*^F^*; *αMHC-Cre*); GLUT1, glucose transporter 1; GLUT4, glucose transporter 4; GAPDH, Glyceraldehyde 3-phosphate dehydrogenase; PGC1α, peroxisome proliferator-activated receptor gamma coactivator 1α; PPARα, peroxisome proliferator-activated receptor α; MCD-1, malonyl-CoA decarboxylase-1; CPT-1β, carnitine palmitoyltransferase-1β; ACC2, acetyl-CoA carboxylase; SCOT, succinyl-CoA-3-oxoacid CoA transferase. CHOP, C/EBP homologous protein; JNK, c-Jun *N*-terminal kinase; PDI, Protein disulfide isomerase; ATF4, activated transcription factor 4; ATF6, activated transcription factor 6; sXBP-1, spliced X-box binding protein-1; *p < 0.05; **p < 0.01; and ***p < 0.001, cKO *versus* CTL.

We examined gene expression profiles regulating metabolism at post-natal day 9. We found significant reductions in the concentrations of glucose transporter 1 (*Glut1*), glucose transporter 4 (*Glut4*), peroxisome proliferator-activated receptor α (*Pparα*), malonyl-CoA decarboxylase-1 (*MCD-1*) and succinyl-CoA-3-oxoacid CoA transferase (*SCOT*) in *Rheb1* mutant mice (Figure 5A,B). Electromicroscopic (EM) analysis revealed dysfunctional mitochondria (Figure 5C) in *Rheb1* mutant mice. Furthermore, TUNEL staining and cleaved caspase 3 detection showed increased cardiomyocyte apoptosis before heart failure in *Rheb1* cKO mice (Figure 5D–G; Supplementary Figure S1). However, no significant autophagy was found in *Rheb1* cKO mice (Supplementary Figure S2).

Accordingly, compromised energy metabolism and increased cardiomyocyte apoptosis may contribute to cardiac dysfunction in *Rheb1* cKO mice.

#### 2.1.5. Knockout of *Rheb1* Results in the Activation of Endoplasmic Reticulum (ER)-Associated Apoptosis Signaling Pathways

The mitochondrial-dependent pathway, death receptor pathway and ER stress-induced pathway are the three major apoptotic pathways in cells. Caspase 12 was activated in *Rheb1* cKO mice at post-natal day 9 (Figure 5H,I), as evidenced by the increased ratio of cleaved caspase 12 to total caspase 12. In addition, a substantial increase in the concentrations of CHOP and p-JNK were observed at post-natal day 9 (Figure 5H,J,K). Subsequently, we checked three sensors of ER (double-stranded RNA-activated protein kinase-like ER kinase (PERK), activated transcription factor 6 (ATF6), and ribonuclease inositol-requiring protein-1 (IRE-1)). We found that the mRNA levels of ATF6 and spliced X-box binding protein-1 (sXBP, indicator of IRE-1 activation) were significantly increased in mutant mice at post-natal day 9 (Figure 5N), whereas activated transcription factor 4 (ATF4, indicator of PERK activation) and other associated ER-stress markers remained unchanged at post-natal day 9 (Figure 5H,L–N; Supplementary Figure S3). Collectively, these results indicate that knockout of *Rheb1* activates ER-associated apoptosis signaling pathways.

#### 2.1.6. Knockout of *Rheb1* Abolishes mTORC1 Signaling

**Figure 6.**
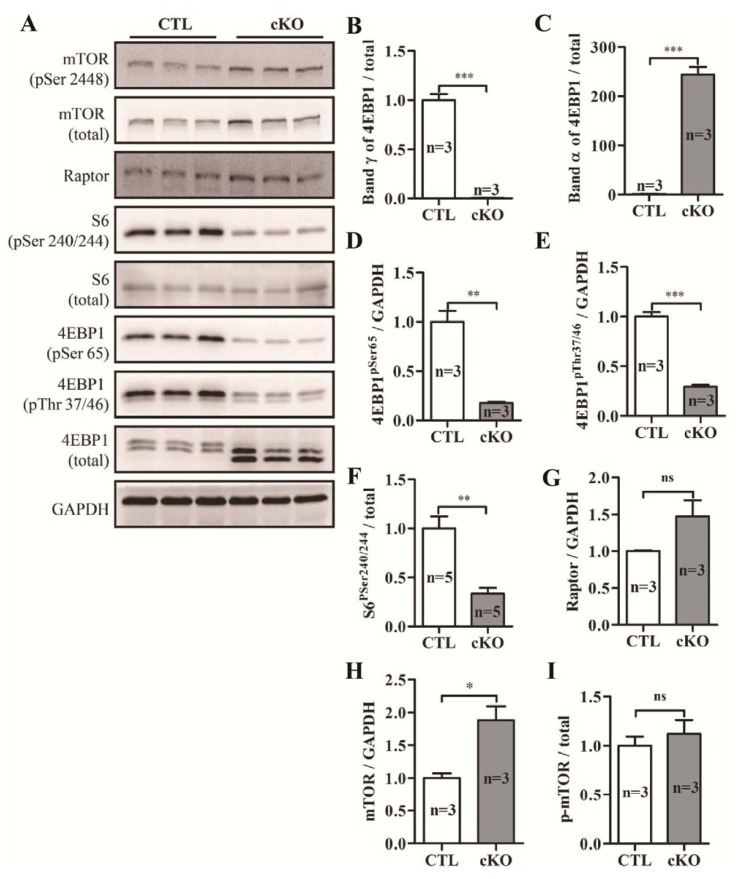
(**A**) Western blot analysis of mTORC1 signaling at post-natal day 9; and (**B**–**I**) Quantification of (**A**) (*n* = 3–5). Abbreviations: CTL, Control group (*Rheb1^F^*^/^*^F^*); cKO, cardiac knockout group (*Rheb1^F^*^/^*^F^*; *αMHC-Cre*); mTOR, mammalian target of rapamycin; Raptor, regulatory-associated protein of mTOR; S6, S6 ribosomal protein; 4E-BP1, eukaryotic initiation factor 4E binding protein 1; *p < 0.05; **p < 0.01; and ***p < 0.001, cKO *versus* CTL.

Phosphorylation levels of S6 ribosomal protein (S6) and eukaryotic initiation factor 4E binding protein 1 (4E-BP1), two downstream targets of mTORC1 signaling, were dramatically reduced *in Rheb1* mutants (Figure 6A,D–F). However, levels of unphosphorylated 4E-BP1 (band α) were significantly increased in *Rheb1* mutant mice (Figure 6A,C). Moreover, levels of mTOR protein and the regulatory-associated protein of mTOR (Raptor, the major component of mTORC1) were significantly increased in *Rheb1* mutants (Figure 6A,G–I).

#### 2.1.7. Removal of *Pten* Prolongs Survival of *Rheb1* cKO Mice

**Figure 7.**
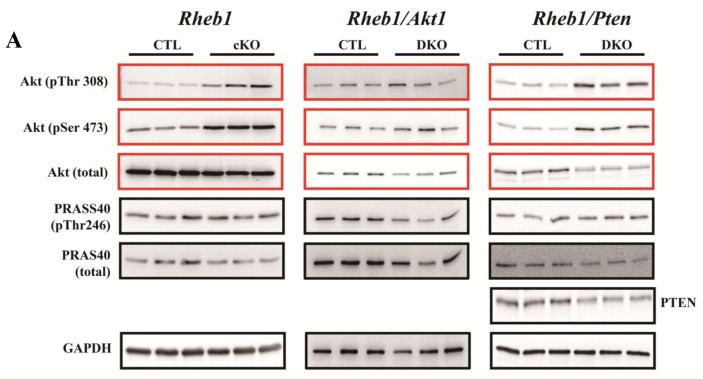

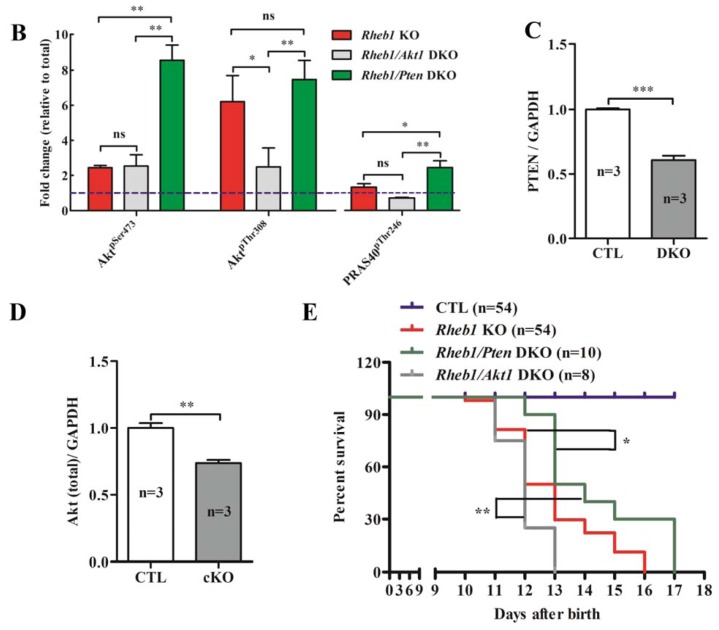
(**A**) Western blot analyses of *Rheb1* cKO and CTL (left panel), *Rheb1*/*Akt1* knockout and CTL (middle panel), and *Rheb1*/*Pten* knockout and CTL mouse hearts (right panel) at post-natal day 9; (**B**) Quantitative comparison of levels of phospho-Akt and phospho-PRAS40 in each panel of (**A**) (*n* = 3); (**C**) Quantification of PTEN in the right panel of (A) (*n* = 3); (**D**) Quantification of total Akt in the medium panel of (A) (*n* = 3); and (**E**) Survival analysis. Abbreviations: CTL, Control group (*Rheb1^F^*^/^*^F^*); cKO, cardiac knockout group (*Rheb1^F^*^/^*^F^*; αMHC-Cre); DKO, double knockout; PTEN, phosphatase and tensin homolog; PRAS40, proline-rich Akt substrate 40 kDa. *p < 0.05; **p < 0.01; and ***p < 0.001.

Compared to CTL mice, *Rheb1* mutant mice exhibited increased levels of phosphorylated Akt at both threonine 308 (T308) and serine 473 (S473). *Rheb1* mutants also showed increased phosphorylation of proline-rich Akt substrate 40 kDa (PRAS40) (Figure 7A,B). Phosphorylation of PRAS40 is an indicator of Akt activity. In order to clarify the role of hyper-phosphorylated Akt in *Rheb1* cKO mice, cardiac-specific *Rheb1*/*Pten* and *Rheb1*/*Akt1* double knockout mice were created to further increase or decrease Akt phosphorylation levels, respectively. Akt phosphorylation was enhanced in *Rheb1*/*Pten* double knockout mice relative to *Rheb1* cKO mice, especially at the S473 site. However, the decrease in p-Akt was only subtle at the T308 site and substrate of Akt was less phosphorylated in *Rheb1*/*Akt1* double knockout mice compared to *Rheb1* cKO mice (Figure 7A,B). Survival analyses revealed that removal of *Pten* significantly prolonged the survival of *Rheb1* cKO mice (Figure 7E). This finding indicates that the activation of the Akt pathway in *Rheb1* cKO mice is a protective adaptive compensatory response.

### 2.2. Discussion

In this study, we showed that *Rheb1* plays an integral role in regulating heart growth, energy metabolism, cardiomyocyte survival, heart function and electrophysiological homeostasis in post-natal mice. Although mouse heart function was maintained without *Rheb1* during the first nine days of life, *Rheb1* cKO mice demonstrated significant changes in heart weight and cardiomyocyte survival, along with affected metabolic gene expression, activated ER-associated apoptosis signaling pathways, and reduced mTORC1 activity during this time period. Moreover, cardiac function began to deteriorate after post-natal day nine, and *Rheb1* cKO mice subsequently developed severe dilated cardiomyopathy. This study thus provides evidence of a causal relationship between loss of function of the *Rheb1* gene and infant heart failure in mice. Equally important, *Rheb1* cKO mice represent a novel animal model of infant heart failure. Furthermore, we showed that *Rheb1* is essential for mTORC1 activation in post-natal mice, and that this signaling pathway promotes cardiomyocyte survival via regulation of ER-associated apoptosis signaling pathways. Meanwhile, activation of Akt signaling helped to improve the survival of infant mice with advanced heart failure. A recent study showed that knocking out *Rheb* in mouse cardiomyocytes at post-natal day three resulted in premature death and dilated cardiomyopathy at post-natal day eight without significant cardiomyocyte apoptosis [7]. In the present study, *Rheb1* cKO mice survived four to seven days longer than in this previous study [7]. In addition, we found that *Rheb1* cKO mice experienced increased cardiomyocyte apoptosis at post-natal day nine before they developed heart failure. While we cannot fully account for these diverging results, it is possible that they stem from differences in the genetic backgrounds of the mice studied in these two research efforts.

Compromised metabolism and abnormal heart growth likely played a significant role in the development of heart failure in *Rheb1* cKO mice. Also, the development of myocardial dysfunction in these mice was mediated by a reduction in mTORC1 activity. Our study provides genetic evidence that *Rheb1* is indispensible for mTORC1 activation in the post-natal mouse heart. We found that *Rheb1* mutant mice experienced abnormal heart growth, retarded cardiomyocyte development and impaired fatty acid oxidation. Signaling through mTORC1 controls heart growth and energy metabolism and is essential for maintenance of normal heart function [8–10]. Thus, it is highly likely that the elimination of mTORC1 activity impairs energy metabolism and cardiac function. Nonetheless, prior work has shown that GLUT1 is upregulated in *Raptor* knockout hearts [9]. Age-related changes in cardiomyocyte metabolism may help to explain these disparate results. Indeed, while our study of *Rheb1* cKO mice involved infant mice, previous work on *Raptor* mutant mice included adult mice. Importantly, while glucose is the primary energy source for the infant mouse heart, the adult mouse heart’s predominant fuel source is fatty acid metabolism [11,12].

Apoptotic cardiomyocytes were increased in *Rheb1* mutant mice, which may be another early trigger of cardiac dysfunction. Previous studies have shown that impaired mTORC1 activity results in increased cardiomyocyte apoptosis [9,10], and that knocking out *Rheb1* causes increased cell apoptosis through non-mTORC1 pathways [13]. However, the precise molecular mechanism by which knocking out *Rheb1* causes apoptosis remains unclear. We found that ER-associated signaling pathways contribute to *Rheb1* cKO induced apoptosis, which was indicated by elevated cleaved caspase 12, CHOP and p-JNK [14–22]. Three ER transmembrane receptors—PERK, ATF6 and IRE1—sense, and initiate apoptosis signaling pathways in response to ER stress [23,24]. PERK-ATF4 and ATF6 can enhance CHOP levels to initiate apoptosis, while IRE1 initiates apoptosis through the IRE1-XBP1-JNK pathway [23,25,26]. We found that ATF6 and spliced XBP1 (sXBP1, indicator of IRE1 activation) were upregulated at post-natal day nine, while ATF4 levels remained unchanged at this time. In addition, Bip and PDI, which are ER chaperones and are involved in processing unfolded proteins, remained unchanged at post-natal day nine. Therefore, we concluded that the ATF6-CHOP and IRE1-JNK pathways contribute to *Rheb1* cKO induced apoptosis. Further studies are required to elucidate how knocking out cardiac *Rheb1* activates ATF6 and IRE1 in *Rheb1* mutant mice, and if activation of ATF6 and IRE1 depends on the mTORC1 signaling pathway.

We also observed hyperphosphorylation of Akt, both at the serine 473 site and threonine 308 site in *Rheb1* cKO mice. This finding is consistent with, and supports, previous work elucidating the S6K-insulin receptor substrate proteins (IRS)-Akt feedback loop [9,10,27,28]. Previous studies have observed lower activity of mTORC1 with Akt hyperphosphorylation in the hearts of humans with advanced heart failure [29,30]. However, the precise role of Akt hyperphosphorylation in the pathogenesis of heart failure has not yet been elucidated in this context. The changes in Akt-mTORC1 signaling pathways (reduced mTORC1 activity with Akt hyperphosphorylation) that we describe in *Rheb1* cKO mice are similar to changes described previously in the hearts of humans with heart failure, suggesting that the *Rheb1* cKO mouse represents an appropriate model for understanding the role of Akt hyperphosphorylation in humans with advanced heart failure. In addition, increased Akt phosphorylation may be protective in *Rheb1* mutant mice [28,31]. Indeed, we observed increased survival of *Rheb1*/*Pten* double knockout mice.

The *Rheb1* cKO mice in this study experienced a number of types of electrocardiographic abnormalities and arrhythmias, including: P wave flattening, conduction block, and ventricular tachycardia. P wave abnormalities occurred earlier than arrhythmias, which could be due to elimination of *Rheb1* in atrial cardiomyocytes before ventricular cardiomyocytes [32]. No significant differences in the incidence of conduction block were observed between *Rheb1* mutant mice and CTL mice. CTL mice may have experienced conduction block because their electrical conduction systems were not yet fully developed, thereby predisposing them to conduction abnormalities when under stress, or exposed to anesthesia. Advanced heart failure may have predisposed *Rheb1* cKO mice to arrhythmias. Alternatively, it is also possible that knocking out *Rheb1* produces an arrhythmogenic substrate via an alternative mechanism [33]. Therefore, further work is necessary to elucidate the precise mechanism(s) by which knocking out *Rheb1* increases the propensity for arrhythmias.

This study has several potential limitations. First, the number of measured samples at post-natal day thirteen was relatively small. This is due to the fact that the majority of *Rheb1* cKO mice died at post-natal day thirteen (median mouse survival was twelve and one-half days). Second, although metabolic genes and ER stress were examined at post-natal day nine—a time when the EF and FS were normal—it is difficult to definitely determine whether the changes in cardiac physiology that we observed in *Rheb1* cKO mice were a direct consequence of knocking out *Rheb1* or were secondary to LV dilatation, which had already begun by this time. Third, the Akt antibody used in this study recognizes all three isoforms of Akt including Akt1, Akt2 and Akt3. Although there were some residue proteins (we think they are Akt2 and Akt3), Akt levels were still significantly lower in *Rheb1*/*Akt1* double KO mice than in CTL mice. In addition, Akt levels were also reduced in *Rheb1/Pten* double KO mice than in CTL mice. This finding is consistent with what we know about the role that PTEN plays in Akt phosphorylation. Indeed, PTEN is a phosphorylase responsible for the transformation of PIP2 to PIP3. This sequence results in increased phosphorylation of Akt. Thus, when PTEN is knocked out—as it is in the *Rheb1/Pten* KO mice—total Akt levels are reduced. Nonetheless, we still do not understand the molecular physiology underlying improved survival rates in *Pten* KO mice. Fourth, we did not investigate why survival differs between *Rheb1/Akt1* KO mice, *Rheb1/Pten* KO mice, and the other mice in this study. Follow up analyses are necessary to further elucidate the mechanisms by which *Rheb1*/*Akt1* and *Rheb1*/*Pten* knock-outs influence mouse cardiac function and survival. Fifth, we did not measure GLUT1 and GLUT4 protein levels. It would also be interesting to measure ATP levels in these groups of mice, as TEM shows compromised mitochondria, while PGC1a levels remain unchanged. Finally, future analysis should also be extended to the *Rheb1/Akt1* and *Rheb1/Pten* KO models in the future as this could mechanistically link the observed phenotypes.

## 3. Experimental Section

### 3.1. Animal Model

Mice of a C57BL/6 genetic background were housed in groups with 12 h dark/light cycles and free access to food. These conditions are in accordance with the Guide for the Care and Use of Laboratory Animals published by the US National Institutes of Health (NIH publication no. 85-23, revised in 1996), and the regulations on mouse welfare and ethics of Nanjing University. All procedures were conducted with approval from the relevant authorities. *Rheb1*-floxed mice were as described previously and were maintained on a C57BL/6 genetic background [4,6]. To knockout *Rheb1* in cardiomyocytes, *Rheb1*-floxed mice were crossed with *α*-myosin heavy chain (*α*-*MHC*)-*Cre* mice and the progenies were genotyped by PCR. DNA primers for genotyping were as follows: *Rheb1* 5′-GCC CAG AACATC TGT TCC AT-3′ and 5′-GGT ACC CAC AAC CTG ACA CC-3′ to amplify wild type (*WT*) and 5′ *floxed* allele. PCR products for *WT* and *floxed* alleles are 650 base pairs and 850 base pairs, respectively (Supplementary Figure S1A).

To generate double-knockout mice of *Rheb1* and *Akt1*, *Akt1^F^*^/^*^F^*; *Rheb1^F^*^/^*^+^*; *αMHC-Cre* mice were crossed with *Rheb1^F^*^/^*^F^*/*Akt1^F^*^/^*^F^* mice. Double-knockout mice of *Rheb1* and *Pten* were obtained in a similar manner.

### 3.2. Echocardiography Assessment of Cardiac Function

Mice received intraperitoneal anesthesia with pentobarbital (70–80 mg/kg). They were monitored by evaluation of the pinch reflex and breathing rate, and situated supine on a warming pad as described in JOVE [6,34]. A Vevo 770 (Visual Sonics, Toronto, Canada), equipped with a 30-MHz transducer, was used for noninvasive transthoracic echocardiography. Two-dimensional guided M-mode tracings were recorded. The internal diameter of the LV in the short-axis plane was measured at end diastole and end systole from M-mode recordings just below the tips of the mitral valve leaflets. The interventricular and LV posterior wall thicknesses were measured at end diastole. LV fractional shortening percentage (LVFS)) and LV ejection fraction (LVEF), two indexes of LV systolic function, were calculated according to guidelines accompanying the Vevo 770 UBM system.

### 3.3. Western Blot Analyses

Western blot experiments were performed as previously described [6]. Heart lysates of mice were prepared in lysis buffer (20 mM Tris, 150 mM NaCl, 10% glycerol, 20 mM glycerophosphate, 1% NP40, 5 mM EDTA, 0.5 mM EGTA, 1 mM Na_3_VO_4_, 0.5 mM PMSF, 1 mM benzamidine, 1 mM DTT, 50 mM NaF, 4 μM leupeptin, pH = 8.0). Samples were resolved by 10% SDS-PAGE and transferred to PVDF membranes (Millipore, Billerica, MA, USA). Membranes were blocked with 5% non-fat milk in TBST (50 mM Tris, 150 mM NaCl, 0.5 mM Tween-20, pH = 7.5) and then incubated with primary antibodies overnight. Antibodies used in this study were purchased from Cell Signaling Technology (CST, Danvers, MA, USA), Santa Cruz Biotechnology Inc., (Dallas, Texas, USA 75220) Bioworld (St. Louis Park, MN, USA), Epitomics (Burlingame, CA, USA), Thermos Scientific (Pittsburgh, PA, USA), NeoMarker (Fremont, CA, USA), and Abcam (Cambridge, MA, USA): Rheb (CST #4935), S6 (CST #2317), phospho-S6 (S240/244) (CST #2215), 4E-BP1 (CST #9452), phospho-4E-BP1 (T37/46) (CST #9459), phospho-4E-BP1 (S65) (CST #9451), total Akt (CST #9272), phospho-Akt (Thr308) (#9275), phospho-Akt (Ser473) (CST #9271), mTOR (CST #2972), phospho-mTOR (CST #2971S), Raptor (CST #2280), JNK (CST #9252), phospho-JNK (T183/Y185) (CST #9251S), Caspase 12 (CST #2202), cleaved caspase 3 (CST #2922), ATF4 (CST #11815), GSK3α/β (CST #5676), phospho-GSK3α/β (CST #9331S), PDI (CST #2446), LC3A/B (CST #4108), PRAS40 (CST #2610S), phospho-PRAS40 (T246) (CST #2997), CHOP (CST #2895), PTEN (CST #9552), α-actinin (sc-15335), GAPDH (#AP0063), cTNT (MS-295-P0), HRP-linked secondary antibodies (Prod #31460 and Prod #31430). Image J software (NIH) was used to perform densitometric analysis (http://rsb.info.nih.gov/ij/).

### 3.4. Histology and Immunofluorescence Staining

The hematoxylin-eosin (H&E), Masson’s staining and immunofluorescence (IF) were performed as described previously [13]. Briefly, heart samples were firstly washed with cold PBS and then fixed in 4% PFA at 4 °C. The samples were processed successively by (a) a 30 min washing in PBS at 4 °C; (b) fifteen minutes of incubation in 30%, 50%, 75%, and 85% ethanol, and then 2 × 10 min of incubation in 95% and 100% ethanol at room temperature (RT); (c) 3 × 10 min of incubation in xylene at RT; (d) Twenty minutes of incubation in paraffin/xylene (1:1) at 65 °C; (e) 3 × 30 min of incubation in fresh paraffin at 65 °C. The processed samples were then embedded in paraffin and sectioned (6 μm thick). The sections were then stained.

Immunofluorescence (IF) staining was performed using anti-cTNT antibody, anti-cleaved caspase 3, and anti-α-actinin respectively at 4 °C overnight. Fluorescence microscopy images were obtained with a Research Fluorescence Microscope (Olympus and Leica) equipped with a digital camera. Images were collected and recorded on an IBM R52 computer using Adobe Photoshop^®^ 5.0.

### 3.5. TUNEL Assay and Transmission Electron Microscopy (TEM) Study

TUNEL assay was performed according to a standard protocol. Briefly, the sections were treated with proteinase K (20 μg/mL) and incubated with terminal deoxynucleotidyl transferase (TdT) and biotinylated dUTP. Prior to performing TEM, mouse hearts were perfused with 2.5% glutaraldehyde in 0.1 mol/L sodium phosphate (pH 7.4), for 10–15 min. After isolating the heart, the heart was cut into small pieces of approximately 2–3 mm, and was then was post-fixed overnight at 4 °C in 2.5% glutaraldehyde in 0.1 mol/L sodium phosphate. After post-fixation, the specimens were processed in the Key Laboratory of Neuroregeneration of Nantong University (Nantong, China).

### 3.6. Quantitative Real-Time PCR for Metabolism Relative Genes and Fetal Genes

Total RNA was extracted from LV myocardium using TRIZOL reagent (Invitrogen, Carlsbad, CA, USA) according to the manufacturer’s protocol. One microgram of total RNA from each specimen was reverse transcribed to cDNA using SuperScript Reverse Transcriptase and random hexamers as primers (Invitrogen, Carlsbad, CA, USA). Quantitative real-time PCR (qRT-PCR) was performed with an ABI Stepone plus instrument (Applied Biosystems, Framingham, MA, USA) using 1× I TaQ SYBR green Supermix Kit (Bio-Bad, Reinach, Switzerland) and 300 nmol/L for forward and reverse primers in a total volume of 20 μL. The mRNA level was based on the critical threshold (Ct) value. *LC3* primers (forward, 5′-GCTGCCTGTCCTGGATAAGA-3′; reverse, 5′-CCTGCGTGGGGTTGAGTTGC-3′) used for real time PCR were designed with the software program Primers Express (Applied Biosystems, Foster City, CA, USA) and synthesized by GenScript Corporation (Nanjing, China). Other Primer sequences for qRT-PCR referred to literature [9]. *Gapdh* was used as an internal control.

### 3.7. Mouse Cardiomyocyte Isolation

Mice were anesthetized with pentobarbital sodium (70 mg/kg ip). The heart was quickly removed from the chest and perfused with Tyroid solution in a retrograde fashion via the aorta at constant pressure (100 cmH_2_O) and speed (3.0 mL/min) at 37 °C for one min. The heart was then perfused for five min with a Ca^2+^ free bicarbonate-based buffer. The heart became swollen and hard, and then it became soft. Approximately seven min later, the left ventricle was quickly removed, cut into several chunks, and further digested in a shaker (60–70 rpm) for ten min at 37 °C in the same enzyme solution. The supernatant containing the dispersed myocytes was filtered into a sterilized tube and gently centrifuged at 500 rpm for one min. After the myocytes were pelleted by gravity for twenty min, the supernatant was aspirated and the myocytes were resuspended in KB solution. Myocytes were stored in HEPES-buffered solution to enable experiments on freshly isolated cells [35].

### 3.8. Statistical Analysis

Results of calculations were presented as means ± SEM. Differences in means between two groups were evaluated with unpaired two-tailed Student *t* tests, and those among multiple groups with one-way ANOVA followed by Bonferroni post-hoc tests. Repeated-measures ANOVA was used to measure cardiac function, body weight, heart weight, and the ratio of heart weight to body weight at multiple points in time. Fisher’s exact test was used for comparisons of the percentage of mice with arrhythmias in the *Rheb1* mutant and control groups. Survival data were analyzed using the log-rank test. All statistics was performed with GraphPad Prism 4.0 software (GraphPad, San Diego, CA, USA). *p* values of <0.05 were considered statistically significant.

## 4. Conclusions

In conclusion, this study provides direct evidence that *Rheb1* has multiple functions in the post-natal mouse heart. In addition to regulating post-natal mouse heart growth and heart function, Rheb1-dependent mTORC1 activation is also responsible for energy metabolism and electrophysiological homeostasis in the mouse heart. Interestingly, knocking out *Rheb1* induces ER-associated apoptosis in mammals’ hearts, a finding that provides new evidence of cross-talk between Rheb1 and ER-stress. Enhancing Akt activity also improves the survival of infant mice with advanced heart failure.

## Supplementary Information
