# Supplementary material for: Cardiac Ablation of Rheb1 Induces Impaired Heart Growth, Endoplasmic Reticulum-Associated Apoptosis and Heart Failure in Infant Mice
Source: Int J Mol Sci. 2013 Dec 13;14(12):24380–98. doi: 10.3390/ijms141224380 (PMC3876117; doi:10.3390/ijms141224380)
Supplement: Supplementary file 1 [file ijms-14-24380-s001.pdf]

Supplemental Information

Table S1. Birth incidence of different genotypes.

Genotype	***Rheb1***$^{F/F}$***; αMHC-Cre***	***Rheb1***$^{F/+}$***; αMHC-Cre***	***Rheb1***$^{F/F}$	***Rheb1***$^{F/+}$
Real value	70 (23.3%)	65 (21.7%)	144 (48%)	156 (52%)
Estimated value	75 (25%)	75 (25%)	150 (50%)	150 (50%)

Table S2. Time course of echocardiography at post-natal day 7, 10, and 12.

**Parameter**	**7**		**10**		**12**	
	**CTL (*n* = 8)**	**cKO (*n* = 8)**	**CTL (*n* = 8)**	**cKO (*n* = 8)**	**CTL (*n* = 5)**	**cKO (*n* = 5)**
IVS;d (mm)	0.33 ± 0.05	0.35 ± 0.06	0.35 ± 0.05	0.31 ± 0.05	0.43 ± 0.03 ‡‡‡	0.34 ± 0.02 *
IVS;s (mm)	0.47 ± 0.09	0.49 ± 0.13	0.53 ± 0.09	0.43 ± 0.09	0.60 ± 0.07 ‡	0.43 ± 0.07 *
LVID;d (mm)	1.70 ± 0.23	1.94 ± 0.14	2.09 ± 0.19 ‡	2.46 ± 0.43 †††	2.07 ± 0.22 ‡	2.68 ± 0.39 **,†††
LVID;s (mm)	0.88 ± 0.32	1.06 ± 0.16	1.05 ± 0.24	1.65 ± 0.58 **,††	1.14 ± 0.19	2.00 ± 0.51 **,†††
LVPW;d (mm)	0.54 ± 0.09	0.49 ± 0.09	0.58 ± 0.09	0.53 ± 0.20	0.59 ± 0.10	0.45 ± 0.11
LVPW;s (mm)	0.82 ± 0.14	0.78 ± 0.16	1.01 ± 0.24	0.74 ± 0.28	0.91 ± 0.19	0.65 ± 0.17
LV Vol; d	8.62 ± 2.92	11.97 ± 2.20	14.37 ± 3.23	22.36 ± 10.49 *,††	14.40 ± 3.73	27.15 ± 9.75 **,†††
LV Vol; s	1.82 ± 1.34	2.53 ± 0.94	2.59 ± 1.63	9.52 ± 8.20 *,††	3.14 ± 1.33	14.02 ± 8.92 **,†††
%EF	81.39 ± 11.95	78.393 ± 8.59	82.41 ± 8.73	63.06 ± 18.68 **,†	78.72 ± 5.46	52.03 ± 14.64 **,†††
%FS	50.00 ± 14.11	45.38 ± 8.35	49.97 ± 9.37	34.28 ± 13.14 *	45.32 ± 5.31	25.99 ± 8.85 *,††
LV Mass	12.81 ± 3.66	16.09 ± 1.88	21.25 ± 6.29 ‡‡	21.79 ± 6.42	22.06 ± 5.30 ‡	23.86 ± 5.78 †
LV Mass Corrected	10.25 ± 2.93	12.87 ± 1.50	17.00 ± 5.03 ‡‡	17.43 ± 5.14	17.65 ± 4.24 ‡‡	20.85 ± 2.80 †

Abbreviation: CTL, Control group (*Rheb1*$^{F/F}$); cKO, cardiac knockout group (α-MHC-Cre/*Rheb1*$^{F/F}$); IVS;d, intraventricular septal thickness in diastole; IVS;s, intraventricular septal thickness in systole; LVPW;d, LV posterior wall thickness in diastole; LVPW;s, LV posterior wall thickness in systole; LVID;d, LV internal diameter in diastole; LVID;s, LV internal diameter in systole; FS, LV fractional shortening; EF, LV ejection fraction; LV Vol; d, LV volume in diastole; LV Vol; s, LV Volume in systole. * $p < 0.05$, ** $p < 0.01$, and *** $p < 0.001$, cKO *versus* CTL. † $p < 0.05$, †† $p < 0.01$, and ††† $p < 0.001$, day 10 and 12 *versus* day 7 respectively in cKO group. ‡ $p < 0.05$, ‡‡ $p < 0.01$, and ‡‡‡ $p < 0.001$, day 10 and 12 *versus* day 7 respectively in CTL group.

Figure S1. Detection of apoptosis. (**A**) Immunofluorescence. Red indicates cleaved caspase 3; green indicates cTNT; blue indicates DAPI staining of nuclei. Original magnification: ×40 (Scale bar = 25 µm). (**B**) Quantification of (A) (n = 3). Abbreviation: CTL, Control group (*Rheb1*$^{F/F}$); cKO, cardiac knockout group (*Rheb1*$^{F/F}$; *αMHC-Cre*). ** $p < 0.01$, cKO *versus* CTL.

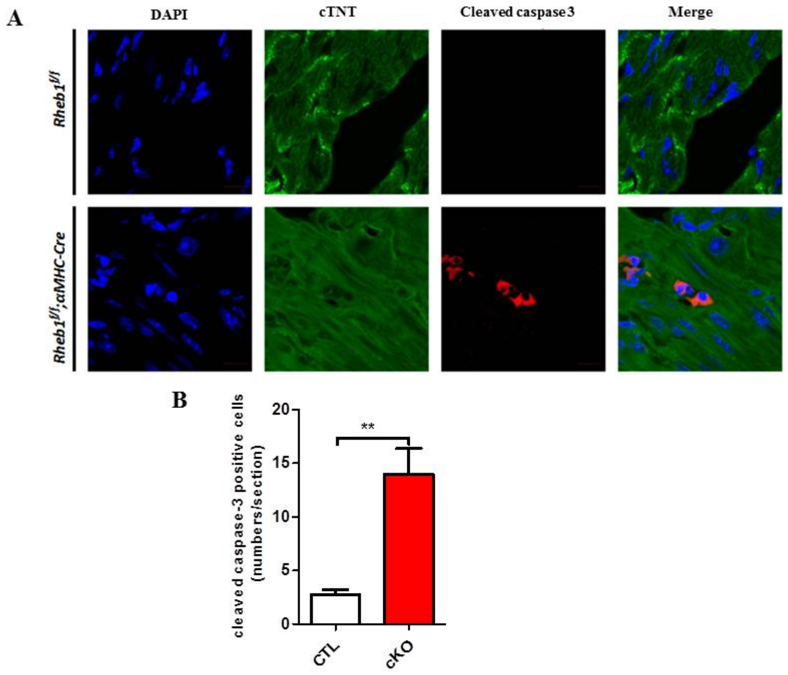

Figure S2. Detection of autophagy at postnatal day 9 and 12, respectively. (**A**) Immunofluorescence. Red indicates LC3A/B; green indicates α-actinin; blue indicates DAPI staining of nuclei. Original magnification: ×40 (Scale bar = 25 µm). (**B**) Representative Western blots of LC3A/B Ⅰ and Ⅱ at postnatal day 9. (**C**) Quantification of (B) (n = 3). (**D**) Detection of LC3 by qPCR (n = 3). (**E**) Immunofluorescence at day 12. Red indicates LC3A/B; green indicates α-actinin; blue indicates DAPI staining of nuclei. Original magnification: ×40 (Scale bar = 10 µm). (**F**) Representative Western blots of LC3A/B Ⅰ and Ⅱ at day 12. Abbreviation: CTL, Control group (*Rheb1*$^{F/F}$); cKO, cardiac knockout group (*Rheb1*$^{F/F}$; *αMHC-Cre*). ** $p < 0.01$; and *** $p < 0.001$, cKO *versus* CTL.

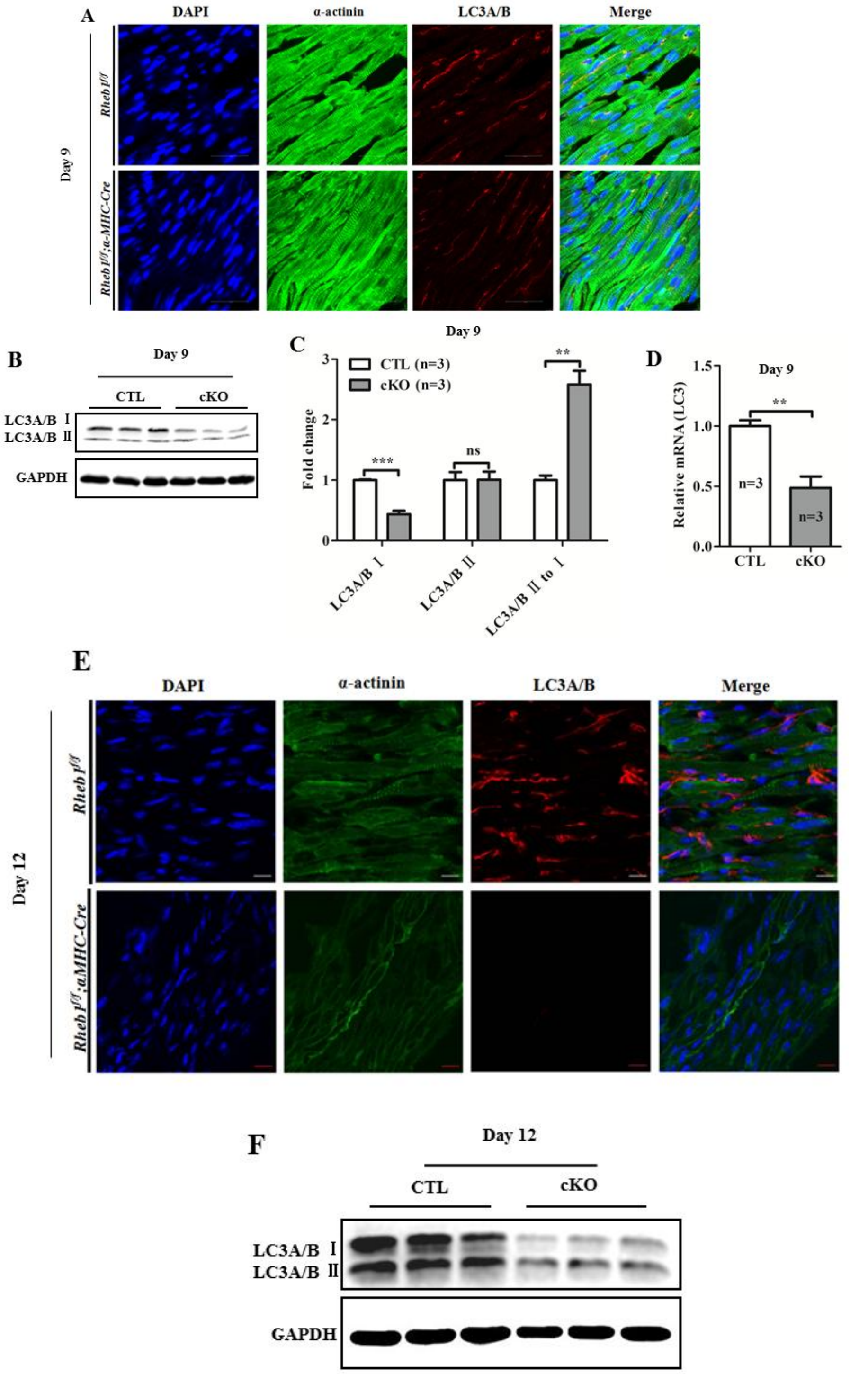

Figure S3. Representative Western blots of ER-stress relative markers at postnatal day 8 and 9 respectively. Abbreviation: ATF4, activated transcription factor 4; PDI, Protein disulfide isomerase; CHOP, C/EBP homologous protein; JNK, c-Jun N-terminal kinase; CTL, Control group (*Rheb1*$^{F/F}$); cKO, cardiac knockout group (*Rheb1*$^{F/F}$; *αMHC-Cre*).

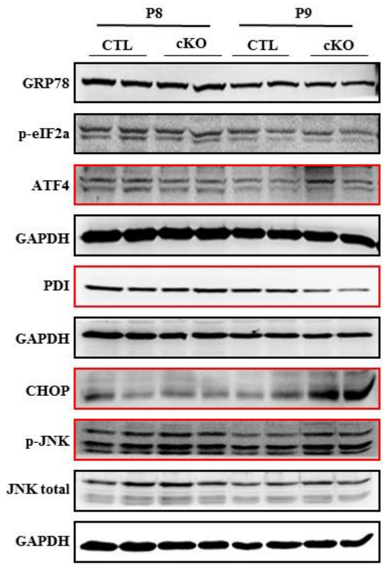

© 2013 by the authors; licensee MDPI, Basel, Switzerland. This article is an open access article distributed under the terms and conditions of the Creative Commons Attribution license (http://creativecommons.org/licenses/by/3.0/).
